# The Potential Involvement of E-cadherin and β-catenins in Meningioma

**DOI:** 10.1371/journal.pone.0011231

**Published:** 2010-06-21

**Authors:** Keiyu Zhou, Guangtao Wang, Yirong Wang, Hanghuang Jin, Shuxu Yang, Chibo Liu

**Affiliations:** 1 Department of Neurosurgery, the Affiliated Taizhou Municipal Hospital, Taizhou University, Taizhou, China; 2 Department of Neurosurgery, Sir Run Run Shaw Hospital, College of Medical Sciences, Zhejiang University, Hangzhou, China; 3 Department of Laboratory Medicine, the Affiliated Taizhou Municipal Hospital, Taizhou University, Taizhou, China; University of Dayton, United States of America

## Abstract

**Objective:**

To investigate the potential involvements of E-cadherin and β-catenin in meningioma.

**Methods:**

Immunohistochemistry staining was performed on samples from patients with meningioma. The results were graded according to the positive ratio and intensity of tissue immunoreactivity. The expression of E-cadherin and β-catenin in meningioma was analyzed by its relationship with WHO2007 grading, invasion, peritumoral edema and postoperative recurrence.

**Results:**

The positive rates of E-cadherin in meningioma WHO I, II, III were 92.69%, 33.33% and 0, respectively, (P<0.05); while the positive rates of β-catenin in meningioma WHO I, II, III were 82.93%, 33.33% and 20.00%, respectively, (P<0.05). The positive rate of E-cadherin in meningioma without invasion (94.12%) was higher than that with invasion (46.67%) (P<0.05). The difference in the positive rate of β-catenin between meningioma without invasion (88.24%) and meningioma with invasion (33.33%, P<0.05) was also statically significant. The positive rates of E-cadherin in meningioma with peritumoral edema 0, 1, 2, 3 were 93.75%, 85.71%, 60.00% and 0 respectively, (P<0.05); the positive rates of β-catenin in meningioma with peritumoral edema 0, 1, 2, 3 were 87.50%, 85.71%, 30.00% and 0 respectively, (P<0.01). The positive rates of E- cadherin in meningioma with postoperative recurrence were 33.33%, and the positive rate with postoperative non-recurrence was 90.00% (P<0.01). The positive rates of β-catenin in meningioma with postoperative recurrence and non-recurrence were 11.11%, 85.00%, respectively (P<0.01).

**Conclusion:**

The expression levels of E- cadherin and β-catenin correlated closely to the WHO 2007 grading criteria for meningioma. In atypical or malignant meningioma, the expression levels of E-cadherin and β-catenin were significantly lower. The expression levels of E- cadherin and β-catenin were also closely correlated with the invasion status of meningioma, the size of the peritumoral edema and the recurrent probabilities of the meningioma, all in an inverse correlationship. Taken together, the present study provided novel molecular targets in clinical treatments to meningioma.

## Introduction

Meningioma originates from the derivatives of meninges and arachnoid cap cells. Its incidence as primary intracranial tumors is very high (15–20%), ranked just behind the cerebral glioma [1], [2]. The biological characteristics of meningioma are diverse. Most of tumors show retarded growth, but a small portion also display invasive growth. Most meningiomas can be cured by complete surgical removal, although there are always the risks of recurrence. The present theory on the occurrence and the development of meningiomas include polygenetic and multiple molecular factors [3], [4]. For example, recent studies suggested that E-cadherin-mediated cell-cell adhesion is critical in these processes [5], [6]. The objectives of this study were to investigate the expression levels of E-cadherin and β-catenin in meningioma with both temporal and spatial information, in order to determine their pathological significance in tumor invasion, formation of peritumoral edema, and postoperative recurrence. The results showed that both the two molecules are tightly associated in meningioma and could have important implications in the development of new targeting therapies.

## Materials and Methods

### Tissue materials

All specimens involved in this study were collected from 49 meningioma patients with university guidelines carefully followed (approved by Taizhou Hospital ethic committe for medical research in using clinical human samples). Written permissions from patients were obtained. These operative specimens came from the Affiliated Municipal Hospital at Taizhou Medical College and Sir Run Run Shaw Hospital at Zhejiang University. All the patients were diagnosed as meningioma in formal pathological reports between Jan 2003 and Sep 2005.

### Clinical data

Eighteen cases were male, and 31 cases were female. The age range was 20 to 75 years, and the average age was 56.3±17.1 years. The length of time for which the patients had meningioma varied from 5 years to 2 days. The first symptoms were as follows: increased intracranial pressure (12 cases), visual disturbance (7 cases), disorder of limb activity (20 cases), hearing disorder (4 cases), seizure (6 cases), and other (10 cases). Cranial MRIs revealed the tumor locations as follows: cerebral hemisphere (15 cases), pardsagittal (17 cases), and cerebellopontine (5 cases) Angle, 6 cases in sphenoid ridge, 6 cases in sellar region; The tumor size was as follows: <3 cm (8 cases), 3∼6 cm (25 cases), >6 cm (16 cases). The dural tail sign was not obvious in 20 cases and was obvious in 29 cases. All 17 cases of pardsagittal tumors had MR venous angiography(MRV), and 2 cases underwent DSA. The results showed that no obstructed sagittal sinus or thrombosis was present. In five cases, the tumors were closely related to the cortical draining veins, but it was easy to separate the tumors and veins during the operation, and there were no obstructions or thrombosis with cortical draining veins. In the samples we studied, 22 cases were Simpson resection grade I, and the other 27 cases were Simpson resection grade II. Invasive tumor was defined according to whether the tumor had invaded the pia mater and skull. This was ascertained by surgical findings and pathological examination. In the invasive group, the tumors had invaded the pia mater or the skull and the arachnoidal cleavage plane had disappeared, while in the non-invasive group, the tumors had not invaded the pia mater or the skull and the arachnoidal cleavage plane was well preserved. According to these criteria, 15 cases were classified as invasive. All of them, the tumor >3cm diameter 2 cases, the tumor 3–6cm diameter 8 cases, the tumor >6 cm diameter 5 cases. Goldman's method was used to classify peritumoral edema [7]. According to this classification method, 16 cases were 0 grade without obvious edema, 21 cases were grade 1 with an edema zone <2cm, 10 cases were grade 2 with an edema zone ≥2cm but restricted to the hemisphere, 2 cases were grade 3 with an edema zone more than the hemisphere. The resected pathological specimens were hematoxylin-eosin (HE) stained. The histological type and grade of the specimens were classified according to the WHO 2000 standard. Forty one cases were benign meningiomas (WHO grade I), 3 cases were atypical meningiomas (WHO grade II), and 5 cases were malignant meningiomas (WHO grade III). Of the benign meningiomas, 10 were epithelial, 5 were transitional, 14 cases were fibrillar, 7 were glit, 2 were angiomas, and 3 were microcystic. Every case included a follow-up visit by the out-patient service or by telephone and letter. The follow-up intervals ranged from 18∼52 months, and the mean follow-up time was 40.9±19.3 months. Upon follow-up, there were 9 relapses, 3 cases had been reoperated, 4 cases had been treated by a gamma knife, and the other 2 cases had been under continuous observation.

### Immunohistochemistry

The expression levels of E-cadherin and β-catenin were measured by immunohistochemical staining and En Vision. Tissues were prepared as paraffin sections. Prior to immunohistochemistry, the sections were deparaffinized with xylene, dehydrated with ethanol, and deoxidised with methanol. The sections were then prepared in a pressure cooker to 121°C for 2 minutes in citrate buffer solution to restore the antigen immunoreactivity. Then the sections were washed in PBS prior to incubation with primary mice monoclonal antibodies of E-cadherin (1∶50, Shanghai Gene Tech Co.) and β-catenin (1∶200, Shanghai Gene Tech Co.) overnight at room temperature. Then the sections were processed for DAB visualization. The sections were then mounted with permount medium and observed under a light microscope.

### Criteria in analyzing the staining pictures

The expression of E-cadherin was located in either the membrane or cytoplasm of meningioma cells, more commonly in the former. The expression of β-catenin was located in the membrane, cytoplasm, and perinuclear granules [8]. The expression strength was analyzed and graded based on the positive ratio and intensity of immunoreactivity [9]. The positive cells were stained light brownish-yellow to chocolate–brown, and the intensity of the immunoreactive products was scored under a high power microscopic as follows: no expression, 0; yellowish, 1; imperial yellow, 2; and brown, 3. The positive ratio was scored as follows: positive cells <5%, 0; positive cells 5–10%, 1; positive cells 11–50%, 2; positive cells 51–80%, 3; positive cells >80%, 4. The two scores were multiplied, and the IRS (values from 0–12) was determined as follows: 0 (−), 1–3 (+), 4–6 (++), and >6 (+++). We selected the best of production quality glass , which had been as observation objects then decided the results of determining.

### Statistical analyses

Statistical analyses were carried out using SPSS 11.0 software. The comparison of the expression strength of E-cadherin and β-catenin with the differentiated pathological types was performed by the rank sum test. The comparison of the positive ratio of E-cadherin and β-catenin with the differentiated WHO 2000 grading, incidence of invasion, level of peritumoral edema, and postoperative recurrence was performed by the Chi-square test. P<0.05 was considered as statistically significant.

## Results

### The expression levels of E-cadherin and β-catenin and their co-relationship to meningioma pathological types (Table 1)

**Table 1:** The expression of E-cadherin and β-catenin with the different pathological types of meningioma.

Pathological type	n	expression of E-cadherin				expression of β-catenin			
		−	+	++	+++	−	+	++	+++
fibrillar	14	3	4	5	2	3	3	4	4
transitional	5	0	3	1	1	1	1	2	1
epithelial	10	0	4	3	3	2	1	3	4
microcystic	3	0	1	1	1	0	1	2	0
angioma	2	0	0	2	0	0	0	2	0
glit	7	0	3	3	1	1	2	3	1
atypical	3	2	1	0	0	3	0	0	0
malignant	5	5	0	0	0	4	1	0	0

We investigated the expression levels of E-cadherin and β-catenin among different types of meningioma. We found that their expression levels were not statistically different between each other (χ^2^ = 5.649, 6.274, respectively; P>0.05). This suggested that E-cadherin and β-catenin could be common molecules participated in the development of diverse meningioma.

### The expression results for E-cadherin and β-catenin with the different pathological grades of meningioma (Table 2)

**Table 2:** The expression levels of E-cadherin and β-catenin in different pathological grades of meningioma.

pathological grade	n	expression of E-cadherin				expression of β-catenin			
		−	+	++	+++	−	+	++	+++
I	41	3	15	15	8	7	8	16	10
II	3	2	1	0	0	2	0	1	0
III	5	5	0	0	0	4	1	0	0

To understand if the amounts of E-cadherin and β-catenin could contribute to the servility of meningioma, we graded the samples of meningiomas following WHO2007 standards. We found that the positive rates of E-cadherin were 92.69%(38/41), 33.33%(1/3), and 0 respectively, for grade I, II, and III (χ^2^ = 28.42, P<0.01). The positive rates of β-catenin for grades I, II, and III were 82.93%(34/41), 33.33%(1/3), and 20%(1/5), respectively, and these differences were significantly different (χ^2^ = 13.09, P<0.05).

### The expression results for E-cadherin and β-catenin with differential invasion of meningioma (Table 3)

**Table 3:** Relationship between E-cadherin and β-catenin expression levels and the aggressiveness of meningioma.

invasion	n	expression of E-cadherin					expression of β-catenin				
		−	+	++	+++	positive rate	−	+	++	+++	positive rate
invasion	15	8	5	1	1	46.67%	10	4	1	0	33.33%
non-invasion	34	2	11	14	7	94.12%	4	5	15	10	88.24%
χ^2^						16.77					11.67
P						0.00					0.00

We further asked if the expression levels of the two proteins could contribute the invasion ability of the tumor. We found significant differences in the expression levels of E-cadherin and β-catenin between invasive or non-invasive meningioma (P<0.05). This strongly suggested that E-cadherin and β-catenin could be potentially negative regulators of the tumor invasion.

### The expression results for E-cadherin and β-catenin in different grades of peritumoral edema (Table 4)

**Table 4:** The expression levels of E-cadherin and β-catenin within different grades of peritumoral edema.

peritumoral edema	n	expression of E-cadherin				expression of β-catenin			
		−	+	++	+++	−	+	++	+++
0	16	1	3	5	7	2	2	2	10
1	21	3	7	10	1	3	4	14	0
2	10	4	6	0	0	7	3	0	0
3	2	2	0	0	0	2	0	0	0

We found that the positive rates of E-cadherin were 93.75% (15/16), 85.71% (18/21), 60%(6/10) and 0, respectively for peritumoral edema graded at 0,1,2,3 (χ^2^ = 11.22, P<0.05). The positive rates of β-catenin for grade 0, 1, 2, and 3 were87.50%(14/16), 85.71% (18/21), 30%(3/10), and 0, respectively (χ^2^ = 17.54, P<0.01).

### The relationship of the expression levels of E-cadherin and β-catenin with the postoperative recurrence of meningioma (Table 5)

**Table 5:** The expression levels of E-cadherin and β-catenin with the postoperative recurrence of meningioma.

recurrence	n	expression of E-cadherin				expression of β-catenin			
		−	+	++	+++	−	+	++	+++
yes	9	6	2	0	1	8	0	0	1
no	40	4	14	15	7	6	9	16	9

Because the expression levels of E-cadherin and β-catenin could reflect the invasive ability of the tumor cells, therefore they might also be implicative for post-operative recurrence. We therefore investigated the association between expression levels of of E-cadherin and β-catenin in our study. We found that the expression levels of of E-cadherin and β-catenin in postoperative recurrence cases were 33.33% and 11.11%, respectively; and for postoperative non-recurrence cases they were 90% and 85%, respectively. In both cases, the differences were significantly different (χ^2^ = 15.49 for postoperative recurrence cases, and 12.84 for postoperative non-recurrence cases, P<0.01).

### The expression results for E-cadherin and β-catenin and the pathological grades of meningioma (Figure 1∼[Fig pone-0011231-g002] [Fig pone-0011231-g003] [Fig pone-0011231-g004] [Fig pone-0011231-g005] 6)

**Figure 1:**
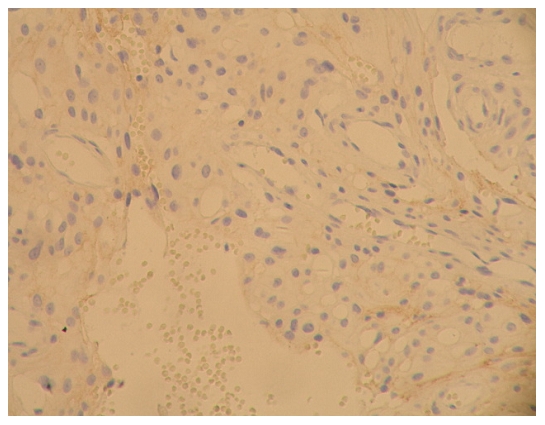
E-cadherin immunostaining on tissue of epithelial type of meningioma (WHO I grade), with amplification of 200X. Expression level was determined as +.

**Figure 2 pone-0011231-g002:**
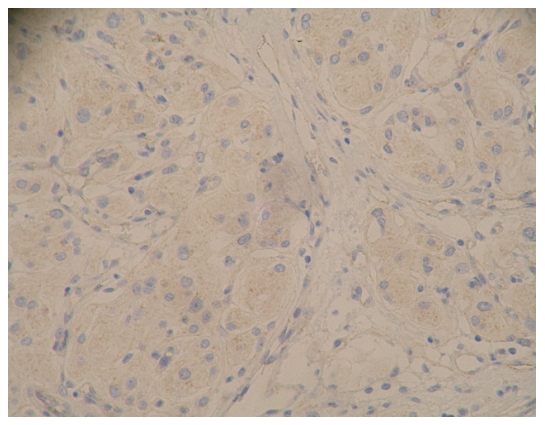
β-catenin immunostaining on tissue of epithelia type of meningioma (WHO I grade), with amplification of 200X. Expression level was determined as +.

**Figure 3 pone-0011231-g003:**
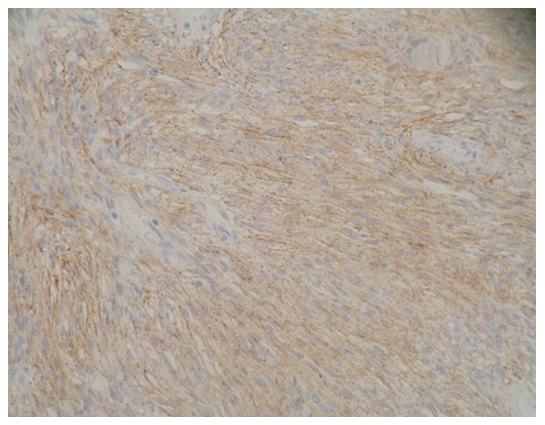
E-cadherin immunostaining on tissue of fibrillar 1 type of meningioma (WHO I grade), with amplification of 200X. Expression level was determined as ++.

**Figure 4 pone-0011231-g004:**
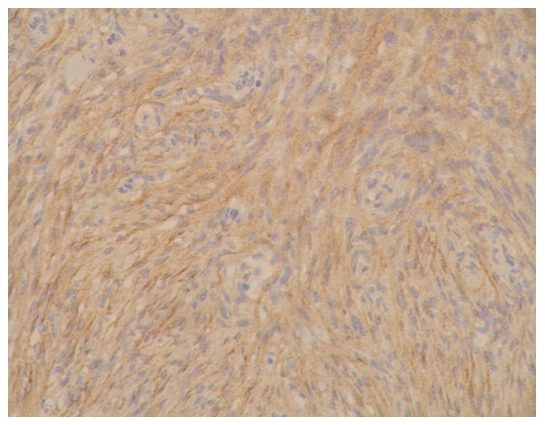
β-catenin immunostaining on tissue of fibrillar 1 type of meningioma (WHO I grade), with amplification of 200X. Expression level was determined as ++.

**Figure 5 pone-0011231-g005:**
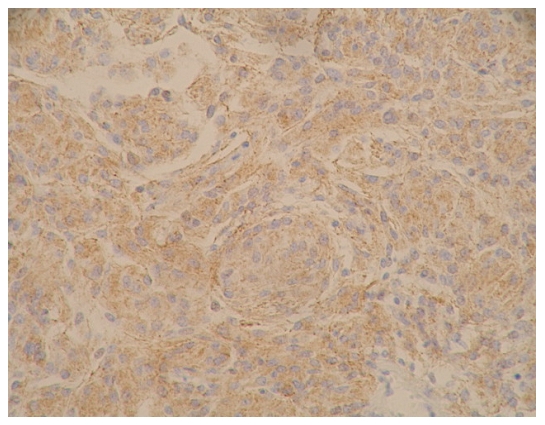
E-cadherin immunostaining on tissue of epithelial type of meningioma (WHO I grade), with amplification of 200X. Expression level was determined as +++.

**Figure 6:**
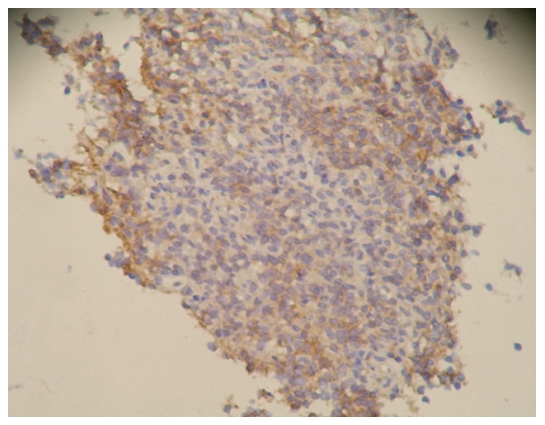
β-catenin immunostaining on tissue of malignant type of meningioma (WHO III grade), with amplification of 200X. Expression level was determined as +.

Figure 1: epithelia (WHO|grade)
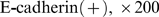



Figure 2: epithelial (WHO|grade)
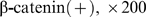



Figure 3: fibrillar l (WHO|grade
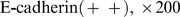



Figure 4: fibrillar l (WHO|grade)
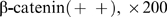



Figure 5: epithelial (WHO|grade)
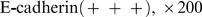



Figure 6: malignant (WHO|grade)
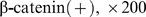



## Discussion

E-cadherin was a calcium-dependent cell-cell adhesion molecule with pivotal roles in epithelial cell behavior, tissue development, and suppression of cancer growth [10], [11]. It was first discovered in 1995 by Berx et al. [12]. Cadherin depended on Ca^2+^ for its function and the structural rigidity; the extracellular amino-terminus formed the “zipper-like” structure with that would act as a cell tight junction. The intracellular carboxyl-terminus of the cadherin molecule indirectly attached the cytoskeleton via catenin. β-catenin, which was one of four known kinds of catenin, is a multifunctional protein [13]. It directly joined to the cytoplasmic terminal of E-cadherin and formed the E-cadherin/catenin complex [14], [15]. Disruption of this junction would lead to diverse phenotypes, including loosed cell-to-cell contacts, morphological changes of the tissue and cells, enhanced cell motility, and the lost of the cell contact inhibition. The changes in molecule structure and function were directly related to the biological behavior of tumor cells, affecting their detachment and re-adhesion.

The expression level of E-cadherin could be related to the classification of astrocytomas. For this reason, an assessment of the expression status of E-cadherin in astrocytomas could be one important index in determining the prognosis of patients [16], [17]. A previous study by Motta et al. examined the expression of E-cadherin in astrocytoma and brain cells with non-CNS tumors [18]. They found that the expression strength of E-cadherin in low-grade astrocytomas (grade I–II) was higher than that presented in high-grade astrocytomas (grade III–IV) (P<0.0001), while the expression strength of E-cadherin in non-CNS tumors is higher than that found in grade I astrocytomas.

The results of our research revealed remarkable differences in the involvements of E-cadherin and β-catenin in different pathological grades of meningioma. Moreover, these differences were statically significant. As the pathological grade of meningioma increased, the positive rates of E-cadherin and β-catenin in meningioma decreased. The expression levels of the E-cadherin and β-catenin were actually completely diminished in malignant meningioma. In previous studies, Utsuki et al. tested specimens of 103 meningioma and found that the expression levels of E-cadherin in 5 atypical meningioma were all negative, 3 cases of the expressions of the β-catenin were negative among them [19]. However E-cadherin and β-catenin expression were positive in epithelial meningioma. In 10 of the 12 cases of invasive meningioma, the E-cadherin and β-catenin expression levels were negative. Therefore they concluded that the decrease in cell adhesion molecules was associated with the increase in tumor cell proliferation and might contribute to invasive ability of meningioma.

Tumor invasiveness and the presence of peritumoral edema were the two major factors that would determine the clinical management of meningioma. There were some biological, physical, and chemical factors that contribute to the peritumoral edema of meningioma [20]. In our studies, we found remarkable differences in the positivity rates for E-cadherin and β-catenin expression corresponding to different degrees of peritumoral edema. As the expression of E-cadherin and β-catenin decreased, possibilities of developing peritumoral edema would increase. We therefore believe that there existed a mechanism in the meningioma cell (even with the high degree of malignancy) which could inhibit the expression of E-cadherin and β-catenin, leading to the harmed cell-to-cell junctions, damaging of the tumor-brain interface and the blood-brain barrier. Consequently, the meningioma cells could infiltrate brain tissue and increase brain edema. In cases that showed a deletion of E-cadherin and β-catenin, one would expect serious brain edema and the clinical features of intracranial hypertension. For this reason, clinical surgeons should pay close attention to the intracranial pressure during a tumor-removal operation.

The decrease or deletion of the expression of E-cadherin leads to the loss of contact inhibition and unrestricted hyperplasia, the loss of intercellular junction with, stronger invasive ability, enhanced tumor cell diffusion and metastasis, as well as benign tumor malignant transformation in some extreme cases [21], [22], [23]. Erdemir F et al. tested specimens of bladder cancers and found that 13 of the 25 stage T1a cases were recurrent and that the positive rate of E-cadherin among them was only 30.7% [24]. However, in the 12 non-recurrent cases, the positive rate of E-cadherin was 75%. Among the stage T1b 27 cases, 25 of the 27 were recurrent, and the positive rate of E-cadherin was only 12%. All these data suggested there was a close relationship between the decreased expression of E-cadherin and recurrence of postoperative bladder cancer. The results of our studies showed that the positive rates of E-cadherin and β-catenin with postoperative recurrence were both significantly lower when compared to those of postoperative non-recurrent cases. We also tested E-cadherin and β-catenin expression levels in pituitary adenoma and found that E-cadherin and β-catenin expression levels were significantly down-regulated and were related to the extent of invasive pituitary adenoma. Pituitary adenoma recurred most easily when the expression of E-cadherin and β-catenin were decreased (Liu et al. Unpublished data).

In summary, the present study employed molecular biology and immunohistochemistry tools to understand the potential involvements of two cell-adhesion molecules in development and invasiveness of meningioma, which provided novel targets for pathological analyses as well as therapeutic drugs.
